# The aeromicrobiome: the selective and dynamic outer-layer of the Earth’s microbiome

**DOI:** 10.3389/fmicb.2023.1186847

**Published:** 2023-05-16

**Authors:** Pierre Amato, Frederic Mathonat, Leslie Nuñez Lopez, Raphaëlle Péguilhan, Zeina Bourhane, Florent Rossi, Jonathan Vyskocil, Muriel Joly, Barbara Ervens

**Affiliations:** Université Clermont Auvergne, CNRS, Institut de Chimie de Clermont-Ferrand (ICCF), Clermont-Ferrand, France

**Keywords:** atmosphere, bioaerosol, aeromicrobiome, bacteria, fungi, cloud

## Abstract

The atmosphere is an integral component of the Earth’s microbiome. Abundance, viability, and diversity of microorganisms circulating in the air are determined by various factors including environmental physical variables and intrinsic and biological properties of microbes, all ranging over large scales. The aeromicrobiome is thus poorly understood and difficult to predict due to the high heterogeneity of the airborne microorganisms and their properties, spatially and temporally. The atmosphere acts as a highly selective dispersion means on large scales for microbial cells, exposing them to a multitude of physical and chemical atmospheric processes. We provide here a brief critical review of the current knowledge and propose future research directions aiming at improving our comprehension of the atmosphere as a biome.

## 1. Introduction

Airborne microbial cell concentrations range from <10 to ~10^7^ cells m^−3^ depending on altitude, location, time of day, and season (Bowers et al., 2011; Tignat-Perrier et al., 2020). The global atmosphere contains ~10^20^ bacteria cells (Whitman et al., 1998), which is approximately 10 orders of magnitude less than in soil and in the oceans, respectively (Šantl-Temkiv et al., 2022). At first sight, such biomass thus seems insignificant, but it is renewed with high turnover (typical particle residence time of a few days) and selectivity (high mortality rates), while providing efficient dispersion at large scale.

Since pioneering visionary scientists investigated the microbes transported in the air [the most prominent of which include (Pasteur, 1862; Molisch, 1922; Meier and Lindbergh, 1935; Moulton, 1942; Lacey, 1986)], aeromicrobiology has emerged as a full-fledged field of environmental microbiology. Over time, it benefited from general technological and knowledge advances in microbiology, and from interactions between disciplines including microbiology, ecology, meteorology, atmospheric physics, and chemistry.

**Figure 1:**
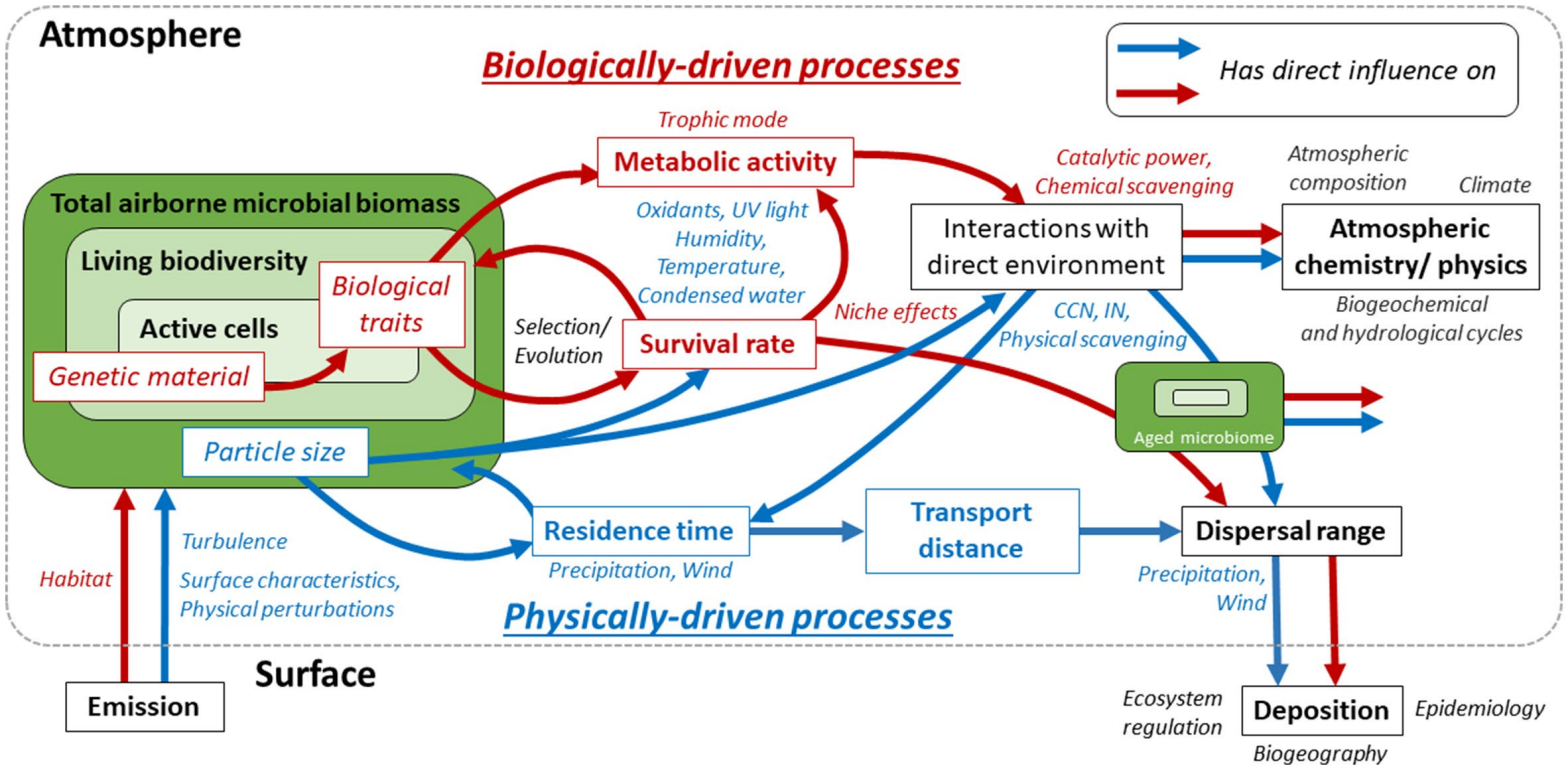
The aeromicrobiome and its identified biological and physical drivers, in red and blue, respectively (arrows and text). Its main intrinsic characteristics are italicized and framed, and major biological and physical factors of regulation are in blue and red italics. Wider hypothesized or proven outcomes, through the processes framed in black, are mentioned in black italics. For example: (i) the survival rate of emitted microorganisms determines the composition of the living aeromicrobiome; it depends on both microorganism’s biological traits, which rely on genetic material, and particle size, and is under pressure from atmospheric conditions (oxidants, UV light, etc.); (ii) microbial cells interact with their atmospheric environment as aerosol particles, with impacts on their residence time (e.g., precipitation), and through metabolic activity, depending on survival and biological traits, which influences chemical processes. More largely, atmospheric composition, hydrogeochemical and hydrological cycles and climate are affected. Aged aeromicrobiome is depicted on the right; it includes a fraction of the emitted cells, and selected viable and active fractions. Once deposited, genetic material and living organisms interact with surface ecosystems, including hosts in the case of pathogens, and contribute to biogeographical and pathogeographical patterns.

The aeromicrobiome demonstrates some level of organization at different scales of space and time, through physical and biological processes as depicted in Figure 1. This contributes to the many aspects of the Earth system, *via* the regulation of ecosystems and populations, hydrological and biochemical cycles, atmospheric composition, climate, biogeography, and microbial evolution. However, despite apparent proximity, the atmospheric biotic system is one of the least studied on the planet and its implications remain still poorly characterized. The selective pressures exerted by atmospheric conditions toward microorganisms depend on physical and biological parameters, and is heterogeneous among the great biodiversity potentially exposed. Some microorganisms remain viable longer than others and, thus, have larger dispersal ranges, while maintaining different degrees of metabolic activity and interaction with their environment. Independently from viability, genetic material is also dispersed and can contribute to large-scale horizontal gene transfer. Microbial cells and biological material also undergo and can affect atmospheric processes, such as cloud formation, precipitation, and chemical reactivity. In this mini-review, we summarize some of the latest developments and key findings related to the atmospheric microbiome, and emphasize major directions for future research.

## 2. Structuration of the aeromicrobiome

### 2.1. Interactions with Earth surfaces

It is largely accepted that anywhere on the planet, the airborne microbial abundance and diversity result exclusively from exchanges with the surface, i.e., emission and deposition processes. At first approximation, emissions and deposition are globally balanced (Burrows et al., 2009b; Fröhlich-Nowoisky et al., 2016), which implies that all aerosolized microorganisms are finally deposited back, without significant production or loss of microbial biomass in the atmosphere, resulting in atmospheric transport as a neutral process. The simplified assumption could be discussed, but it forms the basis of our current view on microbial aerial dispersion. Thus, studying the aeromicrobiome starts by characterizing inputs and losses from and to surfaces.

Aerosolization, i.e., the detachment from surfaces, depends in complex ways on factors such as surface roughness, humidity, and electrical charges, which act against drag forces. Once detached from the surface, particles are lifted by turbulent fluxes (Carotenuto et al., 2017), and their residence time largely depends on particle size and hygroscopicity; these are described in aerosol dispersion models of different resolutions and scales (local to global). However, emission fluxes and dispersion of microorganisms are currently poorly constrained in atmospheric models.

Globally, ~10^24^ bacteria cells are emitted each year from surfaces to the atmosphere (Burrows et al., 2009a), i.e., on average ~ 60 cells/m^2^/s. The aeromicrobiome is highly variable over short spatial and temporal scales (Fierer et al., 2008; Bowers et al., 2011). It has a large species-time relationship compared to, for example, soil or marine environments (Shade et al., 2013), which is directly related to the high turnover (replacement) of taxa (Baselga, 2010; Péguilhan et al., 2021) and the large proportions of rare species (Shade et al., 2014). Its composition and dynamics reflect underlying ecosystem patterns, their spatial extension, and seasonal variations (Bowers et al., 2011; Tignat-Perrier et al., 2020; Archer et al., 2023). The aerial parts of plants notably host a large number of diverse microorganisms that can be readily aerosolized (Vorholt, 2012; Schlechter et al., 2019). Therefore, vegetation acts as a major source of airborne microorganisms with emissions fluxes of up to ~500 viable bacteria cells/m^2^/s measured (Lindemann et al., 1982; Lindemann and Upper, 1985). However, on average, these fluxes rarely exceed ~10 cells/m^2^/s (Carotenuto et al., 2017) and are hence extremely difficult to quantify with accuracy.

Technical and analytical limitations certainly contribute to the current inability to account for microbial emission fluxes. In their current development, these can be experimentally assessed through the Bowen ratio method, approximating that microorganisms’ fluxes above surfaces follow that of sensible heat (Lighthart and Shaffer, 1994). This method involves micrometeorological measurements at high frequency to characterize turbulent fluxes, efficient and accurate sampling devices positioned at 2 heights above ground, e.g., typically ~1 m and 2 m a.g.l. to assess the gradient of microbial abundance (Carotenuto et al., 2017), and the characterization of the surface area “seen” by the samplers and probes, referred to as “footprint” (Schmid, 2002).

In the absence of actual data, emission fluxes are thus to be inferred *a posteriori* based on airborne concentrations measured near the ground, and assuming that removal processes can be appropriately represented. This way, bacteria emission fluxes from major ecosystems were constrained on a global scale by a multivariate approach (Burrows et al., 2009a,b). The underlying assumptions implied underestimated (or ignored) emissions from poorly characterized surfaces, such as oceans, and no temporal variations. The uplift of microorganisms from surfaces is nevertheless highly variable in space and time, in relation for instance with solar irradiance imposing diurnal and seasonal cycles on turbulent fluxes (Lighthart, 1999; Gusareva et al., 2019). Mechanical disturbance of surfaces by wind, rainfall, animals and human activities, and wildland fire can cause efficient aerosolization of microorganisms (Evans et al., 2006; Huffman et al., 2013; Joung et al., 2017; Kobziar et al., 2022). High productivity ecosystems, such as vegetated areas tend to act as long-term net sources (Burrows et al., 2009a), alike the organic-rich microlayer at the ocean–atmosphere interface (sea-surface microlayer) (Aller et al., 2005; Cunliffe and Murrell, 2009; Malfatti et al., 2019; Alsante et al., 2021).

Not all microorganisms have equal chances to enter the aeromicrobiome: those located at the surface-air-interface are inherently more prone to aerosolization than those embedded in complex matrices or deep layers. Therefore, it is conceivable for instance that aerobic organisms are more prone to aerosolization than anaerobes. Some microorganisms have structures designed for aerial dispersion, such as spores in certain fungi, for which emissions can be active processes predictable from meteorological variables (Burns et al., 2022). For other microorganisms, differential aerosolization may occur depending on taxa and their physiological characteristics, some of which can lead to increased buoyancy and flotation (pigmentation linked with cell’ surface hydrophobicity, allometry, …), as shown from aquatic environments using controlled bubble-bursting (Burger and Bennett, 1985; Gauthier-Levesque et al., 2016; Michaud et al., 2018). Microbial biofilms covering Earth surfaces are designed to break up under specific conditions and release free cells, including genetic variants, to colonize distant environments or hosts (McDougald et al., 2012). Aerial dispersion could be integral parts of their life cycle.

Airborne microbial cells finally exit the aeromicrobiome by dry or wet deposition processes, including scavenging by precipitation (Slinn, 1982; Triadó-Margarit et al., 2019; Moore et al., 2020; Péguilhan et al., 2021). Deposition fluxes are much more accessible than those of emission, as deposits can be easily collected and analyzed and directly expressed as per surface area and time. For bacteria, wet deposition is associated with highest fluxes, reaching up to ~10^7^–10^8^ cells/m^2^/h (Reche et al., 2018; Woo and Yamamoto, 2020; Péguilhan et al., 2021). The microbial mixture permanently deposited from air on surfaces brings invaders and competitors to surface environments (Hervas and Casamayor, 2009; Morris and Sands, 2017; Noirmain et al., 2022). The continuous flow of foreign genetic material from atmospheric deposition is hypothesized to contribute to ecosystems stability and microbial evolution (Jalasvuori, 2020).

### 2.2. Microscale distribution

Microorganisms are distributed among aerosol particles in the atmosphere. Size determines the velocity at which particles are removed from the atmosphere by dry deposition, and so their transport range. Settling rates are on the order of ~7 to 750 μm s^−1^ for typical sizes of microbial aerosol particles (0.5 and 5 μm of aerodynamic diameter, respectively, i.e., the equivalent diameter of a perfect sphere of unit density) (Scheuch, 2020); these vary with temperature and pressure. Single bacteria cells with aerodynamic diameter of ~1 μm are estimated to remain airborne for 3–4 days (Burrows et al., 2009a), about twice as short as the residence time of water molecules (van der Ent and Tuinenburg, 2017). This still allows long distance travel over transcontinental scales, as attested for instance by tracers such as Saharan desert dust carrying specific bioaerosols deposited with snow in the Alps, or reaching North America (Creamean et al., 2013; Smith et al., 2013; Barberán et al., 2015; Weil et al., 2017). Such large-scale spread of microorganisms connects distant ecosystems and affects global biogeographical patterns (Morris et al., 2010; Barberán et al., 2014; Leyronas et al., 2018).

The range of sizes of microorganism-carrying particles leads to their zonal separation, horizontally with the distance from emission sources (Gat et al., 2021) and vertically along the altitude (Prass et al., 2021). Very few data exist about the size distribution of microbe-containing particles, that associated with living organisms in particular, or the number of cells and taxa carried together on individual aerosol particles, i.e., the biological mixing state of microbial aerosols, which may affect gene transfer while airborne.

## 3. Aerial fate of microorganisms

### 3.1. Survival of microorganisms during aerial transport

The proportion of living cells, their level of metabolic activity, and the functions expressed are key parameters in the characterization of the aeromicrobiome. A fraction of the emitted microorganisms could already be dead at the time of aerosolization. Moreover, microbial survival is greatly impaired during atmospheric transport due direct exposure to extremely harsh and variable conditions including water availability, temperature, oxidants, and UV radiation (Smith et al., 2011; Joly et al., 2015; Šantl-Temkiv et al., 2022). Most living microbial cells occur as agglomerates larger than 2 μm (Lighthart and Shaffer, 1995b; Lighthart, 1997; Monier and Lindow, 2003). While cell clusters and cells agglomerated to other material have higher survival capacity than single cells, they have a shorter atmospheric residence time; both aspects (particle size and sheltering effect) contribute to the dispersal range of microorganisms. The few available studies assessing the size distribution of microbial particles do not discriminate between viable and total organisms.

Experiments on *Pseudomonas syringae* aerosolized in a cloud chamber determined a half-life time of ~4 h (Amato et al., 2015), indicating that statistically only ~1 individual out of one million survives atmospheric transport from emission to deposition. This is much higher than the mortality rates of bacteria in aquatic ecosystems, on the order of ~10^−3^ h^−1^, mostly driven by predators (Menon et al., 2003). During their atmospheric transport, cells undergo highly selective processes, and the living fraction of microbial aerosols may sharply decrease with time airborne, i.e., increasing horizontal and vertical distance from the emission source. As differential survival capacity exists among the enormous microbial diversity transiting through the atmosphere, aeromicrobiome’s richness and structure may be altered during atmospheric aging.

The proportion of viable organisms is not frequently reported; quantitative data can be difficult to obtain reliably from atmospheric samples due to the necessity to preserve cell integrity and the complex microbial assemblages. Viability is not accounted for by DNA-based techniques, which most current studies rely on. Data derived from cultures represent a conservative underestimate of viability; they typically indicate ~1% viable bacteria, and ~ 50% viable fungal cells (Vaïtilingom et al., 2012). Differential staining (e.g., live/dead staining), a more reliable method, indicated proportions of viable bacteria varying from 2.8 to 6.6% in air sampled from a high mountain site, with higher proportions during the night (Šantl-Temkiv et al., 2017).

Microbial survival rates in relation with environmental variables such as humidity, temperature, particle size distribution, sun radiation, or chemical composition were explored in numerous studies (Ehrlich et al., 1970; Lighthart et al., 1971; Lighthart and Frisch, 1976; Lighthart, 1989; Lighthart and Shaffer, 1997; Tong and Lighthart, 1998; Amato et al., 2015). The survival of bacteria upon water evaporation depends on environmental parameters such as temperature and salinity, and is favored at higher evaporation rates (Marthi et al., 1990; Alsved et al., 2018). In addition, physiological characteristics of taxa are linked with their capacity to maintain viability, including forming resistance spores, pigments, efficient oxidative stress responses and repair systems (Tong and Lighthart, 1998; Ochsner et al., 2000; Fredrickson et al., 2008; Joly et al., 2015). In *E. coli*, series of genes differentially expressed after aerosolization were found to contribute to higher survival, including proteins involved in stress response and DNA protection (Ng et al., 2018). In addition, multiple resistance genes, such as efflux pumps involved in the resistance to quinolones, could provide selective advantage to microorganisms in stressful environments like the atmosphere, even in the absence of such compound, and enhance their capacity to aerial dispersion (Rossi et al., 2022; Smith and King, 2022). *Sphingomonas*, one of the most frequent bacteria taxa in continental atmosphere, often carries multiple resistance genes (Vaz-Moreira et al., 2011).

Overall, some biological traits favoring microbial survival in the atmosphere have been identified. Their selective advantage may vary with atmospheric conditions, i.e., water availability, light radiation, temperature, presence of toxic chemical, etc, and this needs to be better characterized.

### 3.2. Metabolic activity, biological functioning, and potential niche effects in clouds

Biomarkers such as ATP and ribosomal RNA in air and cloud samples indicate the presence of metabolically active bacteria, such as Alpha-Proteobacteria (Rhodospirillales, Sphingomonadales, and Rhizobiales) and Gamma-Proteobacteria (Pseudomonadales) (Klein et al., 2016; Amato et al., 2017; Wirgot et al., 2017; Šantl-Temkiv et al., 2018). This was corroborated by experiments under controlled conditions, in which airborne Alpha-Proteobacteria (*Sphingomonas aerolata*) responded to the presence of volatile compounds by elevating ribosome content (Krumins et al., 2014). Such responses suggest some extent of acclimation to environmental conditions.

The metabolic functioning of airborne microorganisms is expected to vary widely in space and time and with environmental conditions, with potential niche effects in particular in clouds where condensed water could promote biological processes. Only a single study so far demonstrated a general microbial functioning in natural clouds oriented toward the response to stress factors (temperature, oxidants, etc.); metatranscriptomics data indicated series of defense mechanisms associated with central metabolic functions known to participate to stress management (Amato et al., 2019). Microbial multiplication in fog and cloud was suggested based on observations (Fuzzi et al., 1997; Sattler et al., 2001). Further modeling work suggested that significant microbial proliferation is not likely given the short life time of clouds (Ervens and Amato, 2020), but the whole aeromicrobiome functioning could be affected, which still needs to be evaluated.

*In situ* observations of microbial activity in clouds is not possible yet. Enzymatic assays (Vaïtilingom et al., 2010; Qi et al., 2015) as well as chemical fingerprinting of the impacts of microbial activity (Bianco et al., 2019), including isotope-based assays (Sattler et al., 2001), require laboratory incubation. More direct approaches, such as transcriptomics combined with powerful sampling solutions are, thus, preferred. Controlled experiments in simulation chambers might provide further insight into quantitative aspects of microbial activity and its modulations by environmental factors.

Microorganisms are considered specialists or generalists depending on the range of conditions (temperature, salinity, substrates, *etc.*) compatible with their development. Their assembly can be described by niche-driven or neutral (i.e., random) processes, respectively (Liao et al., 2016). Generalists are metabolically more flexible than specialists, resulting in selective advantage in frequently disturbed ecosystems (Chen et al., 2021); these might thus be favored in the atmosphere. The relative abundance of Proteobacteria and Actinobacteria, which include higher proportions of generalists than other phyla, increases with altitude in soils (Luo et al., 2019); these are also frequent in viable airborne assemblages (Vaïtilingom et al., 2012).

The capacity to utilize various sources of nutrients and energy thus seems advantageous for survival and maintenance in the atmosphere. This may contribute to the high abundance of *Pseudomonas* species that are known for their versatility and opportunism (Rojo, 2010). While chemoheterotrophic and photoautotrophic modes are regularly investigated (Amato et al., 2007; Vaïtilingom et al., 2010; Tesson et al., 2016; Dillon et al., 2020), photoheterotrophy, by which cells can harvest additional biochemical energy from light through bacteriochlorophylls and rhodopsins is barely studied. This function may however provide strong selective advantage during atmospheric transport, as suggested by the occurrence of *Sphingomonas*, whose certain species contain anoxygenic phototrophy pigments (Kopejtka et al., 2020). Comparative analyses between the aeromicrobiome and other ecosystems can help deciphering biological functions that provide selective advantage or intimate relation with atmospheric transport, by identifying anomalies in their occurrence.

### 3.3. Impact of microorganisms on the atmosphere

While the previous sections addressed the role of atmospheric conditions for the microbial viability and activity, microbiological processes might, in turn, also affect the atmospheric composition. The role of biological ice nuclei, their distribution in the atmosphere, and their impact on clouds and precipitation have been investigated over the last decade (Hoose and Möhler, 2012; Joly et al., 2014; Pouzet et al., 2017; Patade et al., 2021; Hartmann et al., 2022). Certain plant pathogen bacteria can initiate ice nucleation at higher temperatures than other atmospheric particles. Ice-nucleation active strains of *Pseudomonas syringae* were found at a particularly high frequency in snowfall compared to other environments, and they were identified in feedback processes called “bioprecipitation,” linking vegetation, epiphytic microorganisms, and precipitation (Morris et al., 2008; Bigg et al., 2015). However, to date, the conclusions are not totally clear on the role of ice nucleating bacteria for precipitation (DeMott et al., 2010; Hoose et al., 2010; Burrows et al., 2013; Sahyoun et al., 2017). In addition, chemical, physical, and biological aging processes can modify the ice nucleation ability of biological particles during atmospheric residence time, which makes it difficult to represent them in atmospheric models (Attard et al., 2012; Worthy et al., 2021; Zhang et al., 2021).

Microorganisms have also been suggested to influence the chemical composition of the atmosphere. The biodegradation rates of common cloud-water constituents (e.g., small carboxylic acids) by bacteria were determined in laboratory bulk studies (Vaïtilingom et al., 2013; Bianco et al., 2019; Jaber et al., 2021). Such approaches omitted the fact that cloud droplets are distinct, small environments (~10^−12^ liters), physically spread and exposed to gas uptake, and that biological processes only occur in the small fraction of cloud droplets containing bacteria (statistically <1 out of ~1,000 droplets). Atmospheric model studies found that biodegradation in cloud water may represent a significant sink for water-soluble organic carbon (8 to 11 Tg yr.^−1^), comparable to chemical losses in cloud droplets (Ervens and Amato, 2020; Khaled et al., 2021). The multiple potential impacts of biological activity on the complex atmospheric chemical reactivity should thus be better evaluated and specified.

## 4. Concluding remarks—synthesis of research needs

The atmosphere is a biotic environment. While the need seems obvious to characterize the transport and residence of pathogens for Human, animal, and plant health-related issues, the present review highlights much broader implications of the aeromicrobiome for the planetary health. It is coupled by numerous transport and exchange processes to the Earth surface, the extent of which needs to be better characterized to predict not only the evolution of the aeromicrobiome but also its implications upon deposition.

Future research directions for better characterization of the aeromicrobiome’ organization and its impacts include:

– Characterizing emissions from surfaces: data of airborne microbial concentrations and emission fluxes from surfaces should be collected in particular over vast and poorly characterized areas such as oceans (Burrows et al., 2009b; Hasenecz et al., 2020; Alsante et al., 2021) and forests (Huffman et al., 2012; Crawford et al., 2014), and over sources with potentially important socio-economic impacts, such as agricultural fields and crops (Lindemann et al., 1982; Lighthart and Shaffer, 1995a; Morris et al., 2000; Brunet et al., 2013; Carotenuto et al., 2017). The mass and diversity of microorganisms susceptible to aerosolization has to be constrained for each defined surface category, as well as temporal variations linked with microclimatic and meteorological parameters (turbulence, precipitation, and wind) (Evans et al., 2006), and disturbances such as animal and human activities and wildfires (Kobziar et al., 2022).
– Assessing the dependence of microbial survival, metabolic functioning and activity on atmospheric conditions and biological traits. This includes potential niche effects such as clouds, nutritive conditions, the dependence on particle size, and altitude above ground, along with biological drivers related with trophic modes and biological traits. Size-resolved data of microbial concentration, biodiversity, viability, and functioning should be acquired at defined altitudes from ground, i.e., using towers (Prass et al., 2021), unmanned vehicles (Powers et al., 2018), tethered balloons, or aircraft (DeLeon-Rodriguez et al., 2013), preferably to mountain sites that can be affected by local emissions. Experiments in atmospheric simulation chambers or microcosms can help testing hypotheses. Current basic models of microbial survival rates in aerosol, in relation with environmental variables, should be improved to account for differences between taxa and functions, for implementation in dispersion models (Ehrlich et al., 1970; Lighthart et al., 1971; Lighthart and Frisch, 1976; Lighthart, 1989; Lighthart and Shaffer, 1997; Tong and Lighthart, 1998; Amato et al., 2015).
– Specifying the importance of biological particles for atmospheric processes, and their dependence on atmospheric conditions, e.g., presence of liquid water, temperature, and oxidant levels, which may affect the utilization of nutritive resources as well as cell properties (aging, i.e., destructuration, oxidation of the surface, release of intracellular compounds, *etc.*) (Ballesteros et al., 2001; Bianco et al., 2019). Due to the lack of data, atmospheric model studies are currently limited to the investigation of the biodegradation of a few organic compounds in clouds (Jaber et al., 2020, 2021; Khaled et al., 2021). However, there are indications that similar processes might also occur in the aqueous phase of deliquescent aerosol particles outside clouds. Finally, the aeromicrobiome clearly harbors living organisms interacting with their environment, but the possibility of interactions between airborne microbes (predation, symbiosis, exchange of genetic material, communication, etc.) needs to be elucidated as well.

Such studies will require interdisciplinary efforts by multiple science communities including atmospheric chemistry and physics, meteorology, ecology, microbiology, and also concerted efforts in the development of new measurement and analysis techniques and concepts.

Whether or not the aeromicrobiome can be considered an ecosystem, a mosaic of adjacent ecosystems, or simply a major ecotone extending the boundaries of surface ecosystems and pressuring their microbiomes remains an open question. This environment is still poorly characterized while it may be affected by the rapidly changing conditions on various scales on the planet, with eventual feedbacks stabilizing or aggravating global change trends.

## Author contributions

PA wrote the manuscript with the help and input from all co-authors. All authors contributed to the article and approved the submitted version.

## Funding

This research has been supported by the French National Research Agency (ANR) through the projects MOBIDIC (grant no. ANR-17-MPGA-0013) and METACLOUD (grant no. ANR-19-CE01-0004-02). The authors also acknowledge funding from the Fédération des Recherches en Environnement of Clermont-Ferrand, Clermont Auvergne University, and CNRS.

## Conflict of interest

The authors declare that the research was conducted in the absence of any commercial or financial relationships that could be construed as a potential conflict of interest.

## Publisher’s note

All claims expressed in this article are solely those of the authors and do not necessarily represent those of their affiliated organizations, or those of the publisher, the editors and the reviewers. Any product that may be evaluated in this article, or claim that may be made by its manufacturer, is not guaranteed or endorsed by the publisher.
